# Purulent bacterial pericarditis from *Staphylococcus aureus*

**DOI:** 10.1002/ccr3.2224

**Published:** 2019-05-28

**Authors:** Adam Kaye, Gregory A. Peters, Joshua W. Joseph, Matthew L. Wong

**Affiliations:** ^1^ Department of Emergency Medicine Beth Israel Deaconess Medical Center Boston Massachusetts; ^2^ Harvard Medical School Boston Massachusetts

**Keywords:** echocardiography, infection, pericarditis, tamponade

## Abstract

Purulent pericarditis is a rare condition in the modern antibiotic era. The diagnosis should be suspected in patients with pericardial effusions and radiographic and laboratory investigations consistent with infection. Pericardial fluid culture is the gold standard. Early source control, in addition to antibiotics, is a cornerstone of treatment.

## 1 INTRODUCTION

We present a patient with shock who was found to have cardiac tamponade secondary to a purulent pericardial effusion. After source control and antibiotic therapy, the patient had a full recovery. Purulent pericarditis is rare, and without prompt diagnosis and drainage, the condition can be rapidly fatal.

Bacterial infections of the pericardial space are an uncommon cause of pericardial effusion. Prior to the advent of antibiotics, purulent pericarditis was more common and usually was the result of hematogenous seeding or direct spread from an adjacent infection such as pneumonia. We present the case of a woman who presents in shock with a pericardial effusion, who ultimately diagnosed with MSSA purulent pericarditis and mediastinitis. The diagnosis was suggested by clinical context and cross‐sectional imaging and was confirmed on pericardial fluid analysis and culture. No other bacterial source of infection was found. She responded well to parenteral antibiotics and pericardial drainage and made a full recovery.

## 2 CLINICAL REPORT

A 69‐year‐old woman presented to an outside hospital emergency department (ED) with chest pain, neck pain, back pain, and shortness of breath. She reported fevers, generalized malaise, chills, and sweats. Heart rate was 160 beats per minute, temperature was 97.1° Fahrenheit, blood pressure was 78/52 mm Hg, and respiratory rate was 22 breaths per minute, a saturation of 95% while on room air. She reported that she was in her usual state of health until the previous day, when she developed constant dull pains in her chest, back, and neck. She had no history of IV drug use, and she did not report any other recent illness, nausea, abdominal pains, rash, or leg swelling.

At the outside hospital, she was reportedly ill‐appearing but had clear mentation, and there was no fever, murmur, rash, or swollen joints. EKG demonstrated atrial fibrillation with a rate of 160, normal axis, and normal intervals. Her complete blood count showed a leukocytosis of 27.5 K/μL, a hemoglobin of 10.1 g/dL, and a platelet count of 470 K/μL. A comprehensive metabolic panel was significant for a bicarbonate of 20 mEq/L, a creatinine of 150.28 μ/L, and normal biliary enzymes. A venous lactate was 2.9 Mmol/L. The patient had a positive troponin‐T of 0.10 mg/dL (normal <0.03). PCR testing for influenza was negative. A portable chest X‐ray did not demonstrate pneumonia. Her urinalysis was negative. Blood cultures were not obtained.

At the outside ED, a point‐of‐care echocardiogram demonstrated a small pericardial effusion without right ventricular collapse by report. A femoral triple‐lumen catheter was placed and a norepinephrine infusion was started due to persistent hypotension, and the patient was transiently started on noninvasive ventilation to assist with work of breathing. A CT aortogram of the torso demonstrated small bilateral pleural effusions and a moderate‐density pericardial effusion without aortic pathology, pulmonary embolism, or pneumonia. Imaging of the abdomen and pelvis demonstrated atherosclerotic vascular disease and trace perihepatic fluid of unclear origin. The patient was given vancomycin, piperacillin‐tazobactam, a total of 5 L of 0.9% saline, and she was transferred to our emergency department.

**Figure 1:**
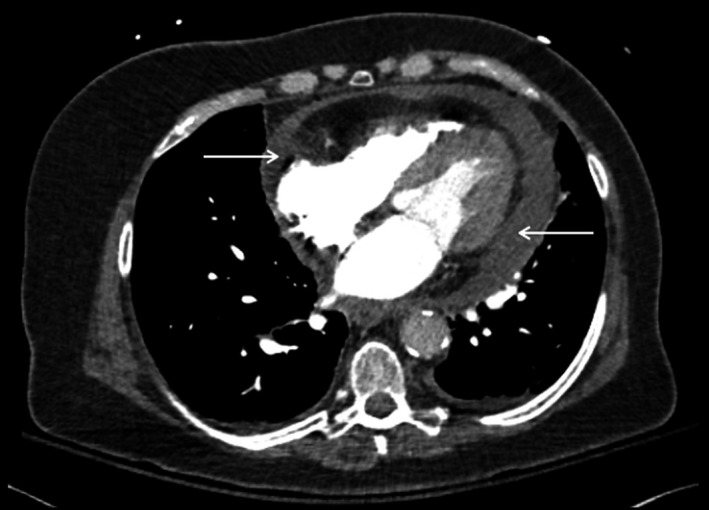
Representative axial image of a contrast‐enhanced CT of the chest, demonstrating a moderately sized moderate‐density pericardial effusion

**Figure 2:**
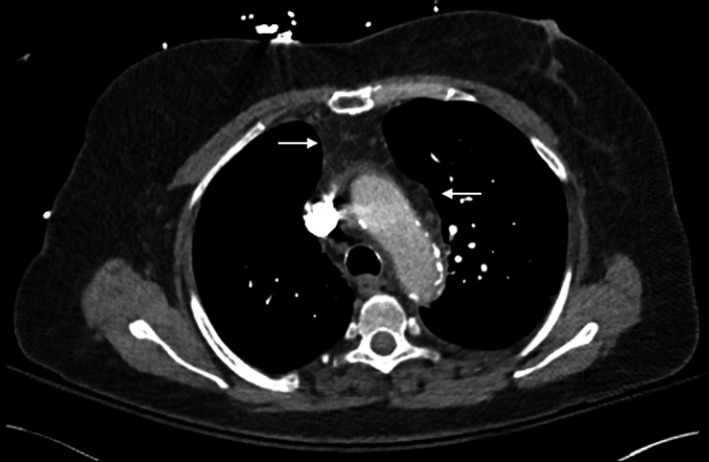
Representative axial image of a contrast‐enhanced CT of the chest, demonstrating subtle mediastinal enhancement

On arrival, the patient had converted to sinus rhythm with a rate of 88 without any ST‐segment abnormalities, the blood pressure was 116/67, temperature was 96.6, and breathing was 18 times per minute with a saturation of 99% while on room air. The rest of her physical examination was unchanged. Further review of the previously obtained CT scan with our emergency radiologists raised suspicion for mild mediastinal stranding and pericardial inflammation (see Figures 1 and 2). A repeat point‐of‐care ultrasound demonstrated a 2‐cm pericardial effusion posteriorly, a 1‐cm effusion anteriorly, no mitral inflow variation with respiration, no right ventricular diastolic collapse, and a plethoric inferior vena cava without respiratory variation. Cardiology and thoracic surgery were consulted for the evaluation of possible septic pericardial effusion and tamponade. The patient was then admitted to the medical intensive care unit for further investigation and management.

**Figure 3:**
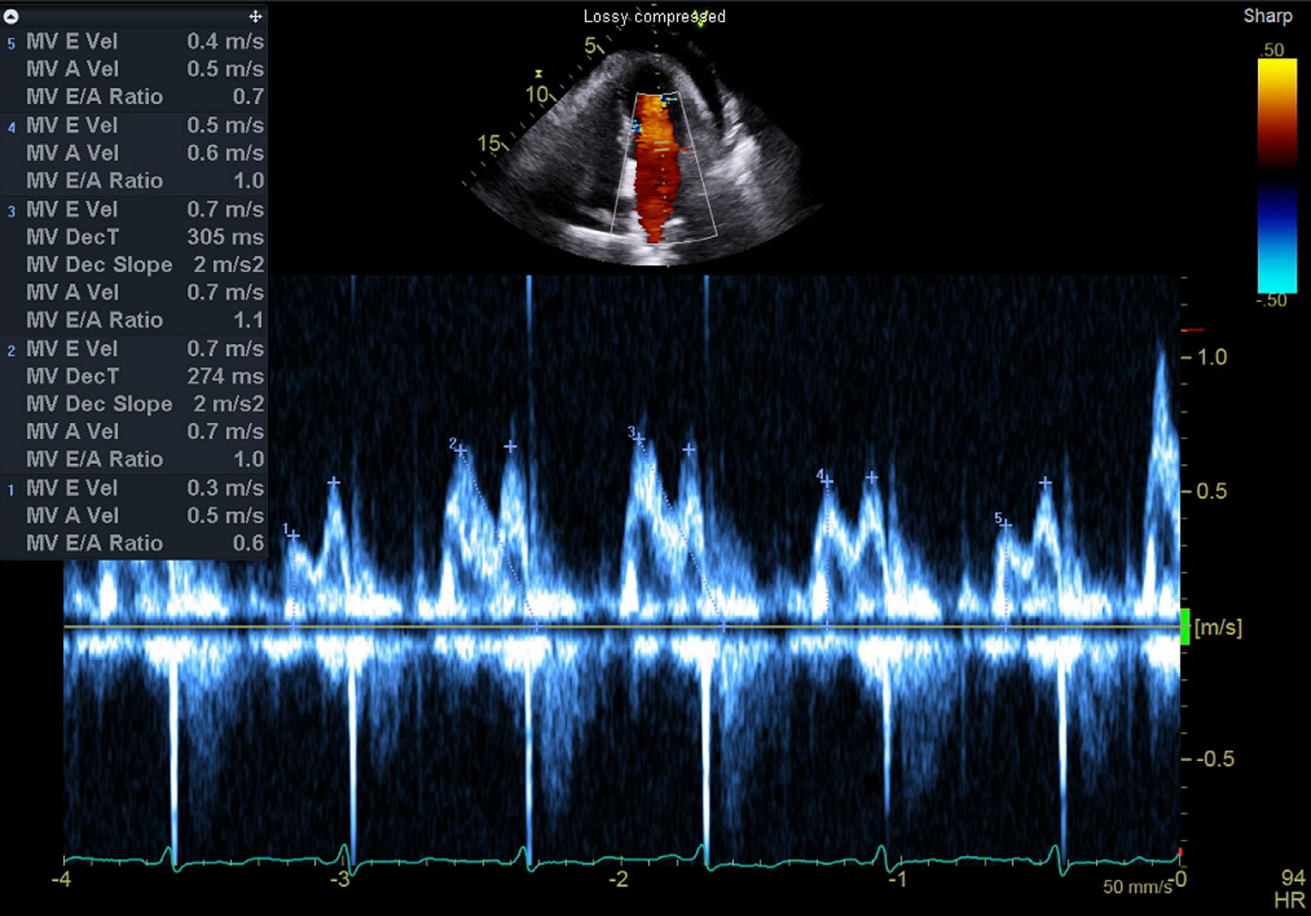
Pulsed Doppler echocardiography of blood entering the left ventricle from the left atrium, acquired from the apical four‐chamber position. Each pair of spikes represents inflow during diastole, the preceding representing early passive diastolic filling, the later contributed by atrial contraction. Between the second and the sixth complexes, early‐diastolic mitral inflow velocities measure 0.3, 0.7, 0.7, 0.5, to 0.4 m/s, respectively

Throughout the following day, her clinical status worsened and a comprehensive echocardiogram demonstrated right ventricular diastolic collapse and accentuated mitral inflow variation, consistent with pericardial tamponade (Figure 3). The patient was taken to the catheterization laboratories, and the pericardium was entered under ultrasound and fluoroscopic guidance, with initial pericardial pressure measuring 15 mm Hg. A pericardial catheter was placed, 320 mL of straw‐colored fluid was removed, and after drainage, the pericardial pressure was zero. The patient's shock resolved with pericardial drainage, and the fluid analysis demonstrated 12 507 nucleated cells/µL with 86% polymorphonuclear predominance and 9629 red blood cells/µL. The pericardial fluid had a glucose of 67 mg/dL, and the Gram stain was negative. On the second hospital day, the pericardial fluid grew Gram‐positive bacteria ultimately determined to be *Staphylococcus aureus*. Repeat echocardiograms demonstrated resolution of the effusion without valvular vegetations, and the pericardial drain was removed on the third hospital day. The patient's blood cultures did not grow any bacteria. Infectious disease, thoracic surgery, and cardiology concluded that the patient had a purulent MSSA pericarditis of unclear etiology, with reactive mediastinitis and recommended a 4‐week course of parenteral cefazolin. The patient followed up in outpatient infectious disease and cardiologic clinics and recovered well.

## 3 DISCUSSION

Purulent pericarditis is a rare, life‐threatening infection of the pericardial space that may initially elude diagnosis. Purulent pericarditis was more prevalent in the pre‐antibiotic era and was a feared complication of pneumococcal pneumonia.^1^ Although purulent pericarditis occurs in only an estimated 1 of 18 000 hospitalized patients, the progression can be very rapid, and half of all cases are diagnosed on autopsy.^2^ The pericardium lacks compliance, and an expanding bacterial infection can rapidly increase pericardial pressures and cause tamponade.

Aside from pulmonary parenchymal disease, purulent pericarditis is associated with other infections of the mediastinum and neck, such as empyema, mediastinitis from dental infection, intracardiac medical devices, eroding intrathoracic malignancies, and endocarditis and bacteremia.^1^, ^3^, ^4^, ^5^, ^6^, ^7^, ^8^, ^9^, ^10^, ^11^, ^12^, ^13^, ^14^, ^15^, ^16^, ^17^, ^18^ The microbiology of purulent pericarditis reflects this, with *Staphylococcus*, *Streptococcus*, *Haemophilus*, and *Mycobacterium tuberculosis* predominating, though atypical organisms such as *Candida* and *Salmonella* have been described. Polymicrobial infections are uncommon.^3^, ^19^ Dialysis, thoracic surgery, chemotherapy, immunocompromise, and AIDS are all risk factors for purulent pericarditis.^3^

The presenting symptoms and initial laboratory blood tests of purulent pericarditis are not specific for pericardial infection. Fever is the most frequent presenting sign (85%), while only half of cases will have an EKG abnormality consistent with pericarditis, and less than a third present with chest pain or a friction rub on auscultation.^6^ Chest X‐rays often are abnormal, demonstrating subtle signs of cardiomegaly, mediastinal widening, pulmonary infiltrates, or pleural effusions. Echocardiography can identify pericardial fluid, as well as pleural fluid or vegetations on cardiac valves. But the diagnosis can only be confirmed by pericardial fluid inspection and culture. Aspiration of pus or positive bacterial culture of pericardial fluid is considered definitive. While infected pericardial fluid usually has a low glucose and high white blood cell count, fluid analysis is not sufficient to differentiate rheumatological from infectious causes.^3^, ^20^

This diagnosis is challenging and requires a high degree of suspicion. Treatment should be initiated promptly with broad‐spectrum antibiotics and source control. In some case series, pericardiocentesis catheters are used to instill fibrinolytics into the pericardial space, which facilitate the clearance of loculations, and DNase may be useful as well.^20^ Only a third of patients can be managed with a pericardiocentesis alone, and most will require surgical pericardial resection or pericardectomy or another surgical drainage.^6^ Antibiotic therapy without source control is unlikely to be beneficial. Outcomes are favorable when treated appropriately, but when left untreated the mortality approaches 100%.^3^, ^6^

## 4 CONCLUSION

Purulent pericarditis is a rare condition that most physicians probably have not encountered before. The pericardium is inelastic and rapid accumulations of fluid, infected or otherwise, rapidly increase pericardial pressures and can cause hemodynamic collapse. As with all pericardial effusions, diligent hemodynamic monitoring and echocardiography should inform care. As with all infection, source control either by pericardiocentesis or surgical technique needs to be employed early.

## CONFLICTS OF INTEREST

None declared.

## AUTHOR CONTRIBUTIONS

AK, GAP, JWJ, and MLW: contributed to the creation and review of this manuscript.
